# Psychopathology and Continuous Subcutaneous Insulin Infusion in Type 1 Diabetes

**DOI:** 10.1155/2013/672729

**Published:** 2013-09-19

**Authors:** Francesco Rotella, Caterina Lamanna, Ilaria Dicembrini, Carlo Faravelli, Caterina Calasso, Edoardo Mannucci

**Affiliations:** ^1^Diabetes Agency, Careggi Teaching Hospital, Via delle Oblate 4, 50141 Firenze, Italy; ^2^Department of Health Sciences, University of Florence, Viale Pieraccini 6, 50139 Firenze, Italy

## Abstract

*Aim*. Continuous subcutaneous insulin infusion (CSII) is used as an option in patients with diabetes failing to multiple daily injections (MDI). Psychological factors may play a relevant role in the failure to attain therapeutic goals in patients on MDI. This could lead to an overrepresentation of psychopathology in patients treated with CSII. *Methods*. A consecutive series of 100 patients with type 1 diabetes was studied, collecting main clinical parameters and assessing psychopathology with the self-reported questionnaire Symptom Checklist 90-revised. Patients on CSII were then compared with those on MDI. *Results*. Of the 100 enrolled patients, 44 and 56 were on CSII and MDI, respectively. Among men, those on CSII were younger than those on MDI; conversely, no difference in age was observed in women. Women on CSII showed higher scores on most Symptom Checklist 90 subscales than those on MDI, whereas no differences were observed in men. *Conclusion*. Women with type 1 diabetes treated with CSII display higher levels of psychopathology than those on MDI. This is probably the consequence of the fact that patients selected for CSII are those failing to MDI. Higher levels of psychopathology could represent a limit for the attainment and maintenance of therapeutic goals with CSII.

## 1. Introduction

Continuous subcutaneous insulin infusion (CSII) is considered a therapeutic option for patients with type 1 diabetes inadequately controlled on multiple daily injections (MDI), producing beneficial effects on glucose control and quality of life [1]. However, in systematic reviews and meta-analyses of randomized trials the improvements associated with CSII are usually very small [1–3]; in the few available trials in type 2 diabetes, the efficacy of CSII does not even seem to differ from that of MDI [4]. On the basis of those results, some authors are inclined to think that the additional benefits provided by CSII could be limited [1]. Those opinions can affect the decisions of authorities, which often limit the reimbursement of expensive devices unless the clinical benefits are clearly proven [5].

On the other hand, there are some theoretical reasons which suggest that randomized clinical trials could underestimate the potential benefits of CSII. First of all, patients enrolled in the clinical trials on CSII are usually already insulin-treated, those were allocated to CSII switch from MDI to CSII, and they could need some time to adapt to their new treatment strategy. Furthermore, available trials on CSII in type 1 diabetes are performed on patients who do not reach a satisfactory glycemic control with traditional insulin therapy; therefore, their characteristics may differ from the average of individuals with type 1 diabetes. In other terms, it is possible that those considered for CSII trials are more resistant to any insulin treatment. In particular, a higher level of psychopathology, leading to unsatisfactory glycemic control with MDI, could be associated with CSII. In fact, psychological factors play a relevant role in the attainment and maintenance of glycemic targets, and psychopathology is recognized as a common cause of unsatisfactory blood glucose control [6]. The present survey was designed to verify this hypothesis.

## 2. Materials and Methods

We studied a consecutive series of 100 outpatients with type 1 diabetes referred to the Outpatient Diabetes Clinic of Careggi Teaching Hospital in Florence, Italy, between September 3rd 2012 and October 26th 2012. Diagnosis of type 1 diabetes was based on clinicians' judgment. Patients with illiteracy and/or cognitive impairment, to prevent the compilation of the self-reported questionnaire (see below), were excluded as well as those who were not familiar with the Italian language. Two subjects were excluded because of the inadequate knowledge of the language. The study was approved by the local Ethics Review Board. All participants provided their informed consent prior to the enrolment.

Data on diabetes duration, concurrent treatments, and associated medical conditions were collected from patients' clinical records, whereas HbA1c was measured with an HPLC method (IFCC standard, Biorad, Hercules, CA, USA) within 7 days from the visit. Furthermore, psychopathology was assessed during a routine clinic visit by means of the self-reported questionnaire Symptom Checklist 90-revised (SCL-90-R) [7], a psychometric instrument devoted to the identification of the psychopathological distress. This psychometric test provides scores for different psychopathological areas (e.g., anxiety, depression, obsessive-compulsive symptoms, etc.), without predefined pathological thresholds for each scale. As a consequence, SCL-90 measures psychopathology as a dimension, but it does not allow formal categorical diagnoses. Only questionnaires with over 90% of completed items were considered valid for analysis.

Data were summarized as mean ± SD if normally distributed, otherwise they were summarized as median (quartiles). Scores of SCL-90 were assumed to be not normally distributed. Comparisons across groups were performed using Student's *t*-test or Mann-Whitney *U* test whenever appropriate. Those comparisons were performed separately for gender, considering the differences in psychopathology between men and women in the general population [8]. The Statistical Package for the Social Sciences for Windows SPSS (IBM, 2011) version 20.0 was used for data analysis.

## 3. Results and Discussion

### 3.1. Results

Of the 100 enrolled patients (61 and 39 women and men, resp.), 27 were affected by diabetic retinopathy and 14 by microalbuminuria. In addition, 4 patients complained of symptomatic peripheral diabetic neuropathy, whereas no patient reported diabetes-related visual impairment, symptomatic arteriopathy of lower limbs, or previous major cardiovascular or cerebrovascular events. Thirteen patients were also receiving treatment with metformin: 12 were treated with ACE inhibitors or angiotensin receptor blockers and one with acetylsalicylic acid. None of the patients reported a history of psychiatric disorders, and none was treated with psychotropic medication, except two women that reported the use of low-dose benzodiazepines.

Among those 100 patients, 44 and 56 were on CSII and MDI, respectively. All patients on CSII had been previously treated with MDI. All patients on CSII except 3 had switched from MDI to CSII more than 3 months before enrolment.

The characteristics of patients within each treatment group are reported in Table 1. Among men, those on CSII were significantly younger than those on MDI, whereas duration of diabetes and HbA1c did not differ across groups.

All enrolled patients completed over 90% of items of SCL-90. Overall, SCL-90 total and subscale scores were not significantly different between women and men (data not shown); conversely, women on CSII showed higher scores on most SCL-90 subscales, whereas no differences between CSII and MDI were observed in men.

### 3.2. Discussion

The present survey suggests that, at least among women, patients who are treated with CSII display higher levels of psychopathology than those on MDI. Although a cross-sectional study does not allow any causal inference, it seems very unlikely that CSII, which has been associated with higher treatment satisfaction and improved quality of life [1, 9], produces psychopathology. On the other hand, patients with psychological disturbances, who may experience greater difficulties in reaching and maintaining an adequate glucose control, could have a greater chance of being treated with CSII. In fact, a previous study had reported a higher prevalence of depressive symptoms in patients on CSII [10] without exploring other psychopathological areas. In the present survey, patients treated with CSII had higher scores on multiple areas (e.g., anxiety, depression, and obsessive-compulsive symptoms), suggesting a difference in overall psychopathology, rather than a specific drive related to depression. The lack of significant differences in men could be due either to a lower prevalence of psychopathology in the male gender (which was not observed in the present sample) or to a greater impact of psychological disturbances on diabetes management and control in females.

The differences in psychopathology can have a relevant prognostic impact. There is wide evidence that mental disorders (e.g., depressive disorders and eating disorders) are associated with impaired glycemic control in patients with type 1 diabetes [11]; furthermore, the presence of psychopathological symptoms, even without a full-blown psychiatric disorder diagnosis, can be sufficient to affect glucose control [11]. If patients on CSII have a higher psychopathology, they should also be expected to encounter greater difficulties in attaining (and maintaining) therapeutic targets. Psychological problems and mental disorders are often cited as barriers to treatment adherence [6]. However, it is also possible that some psychological disturbances affect glucose control through different mechanisms, such as the activation of hormonal or inflammatory pathways [12].

Another interesting finding is that men on CSII are younger than those on MDI, whereas this difference is not evident among women. It can be speculated that the use of newer technologies is easier for younger individuals, as observed for smartphones or other digital devices [13]. In this perspective, the younger age of men on CSII is not surprising. On the other hand, the fact that such difference is not observed in women may depend on other factors. In particular, it is possible that younger women experience grater discomfort in using a device which may be perceived as a limitation in exposing one's body.

Some limitations of the present study should be recognized. First of all, as already stated above, the cross-sectional design does not allow any causal inference. Furthermore, the size of the sample is limited, preventing the detection of smaller differences across groups. The sample was composed of patients referred to a main academic facility for the treatment of diabetes, who cannot be considered representative of individuals with type 1 diabetes in the general population; in fact, the proportion of those on CSII was markedly higher than that (about 4%) reported for the whole country [14]. In addition, the study was monocentric; for this reason, the allocation of patients to CSII could reflect local, rather than general, attitudes. A further limitation is represented by the fact that psychopathology was assessed only by the means of a self-reported questionnaire; although this instrument has been widely validated, its reliability cannot be compared to that of interviews. For this same reason, it was not possible to formulate psychiatric diagnoses using DSM-IV-R criteria [15]. Despite these limitations, the observation that psychopathology differs in those using CSII is potentially interesting, and it deserves to be further investigated in larger samples.

## 4. Conclusion

The fact that patients addressed to CSII, being failures to MDI, have a higher psychopathology than average individuals with type 1 diabetes should be taken into account when assessing the effects of treatment with CSII. In fact, psychological disturbances could be a major factor leading to inadequate treatment response, preventing the attainment of therapeutic targets.

## Figures and Tables

**Table 1:** Characteristics of patients on CSII and MDI.

Characteristics	Women		Men	
	CSII	MDI	CSII	MDI
Number	27	34	17	22

Age (years)	39.8 ± 14.5	39.4 ± 10.7	37.0 ± 9.5*	43.3 ± 8.9
Duration of diabetes (years)	16 [11; 25]	18 [11; 25]	12 [7; 16]	10 [7; 14]
HbA1c (%)	7.4 [7.2; 7.7]	7.5 [6.8; 8.1]	7.6 [7.3; 7.7]	7.4 [7.1; 7.8]
(mmol/mol)	57 [55; 61]	58 [51; 65]	60 [57; 61]	58 [54; 62]

SCL-90 GSI	2.2 [1.3; 2.3]**	1.6 [0.9; 2.1]	2.0 [1.5; 2.2]	1.5 [1.2; 2.1]
SCL-90 PST	86 [72; 89]*	75 [54; 85]	81 [70; 88]	77 [60; 87]
SCL-90 PSDI	2.3 [2.0; 2.4]**	2.0 [1.6; 2.3]	2.2 [1.9; 2.3]	2.0 [1.7; 2.2]
SCL-90 Somatization	1.8 [1.2; 2.0]	1.6 [1.2; 1.7]	1.5 [1.2; 1.7]	1.5 [1.2; 1.8]
SCL-90 Obsessive-Compulsive	2.1 [1.4; 2.5]**	1.4 [0.8; 2.0]	1.8 [1.2; 2.2]	1.4 [0.9; 2.1]
SCL Interpersonal Sensitivity	2.4 [1.6; 2.7]*	1.9 [1.1; 2.4]	2.1 [1.6; 2.6]	1.8 [1.4; 2.4]
SCL-90 Depression	2.5 [1.7; 2.8]**	2.1 [1.4; 2.5]	2.4 [2.0; 2.5]	2.0 [1.6; 2.5]
SCL-90 Anxiety	2.2 [1.1; 2.3]**	1.3 [0.9; 1.9]	1.9 [1.4; 2.1]	1.5 [1.1; 1.9]
SCL-90 Anger/Hostility	2.3 [1.5; 2.7]*	1.8 [1.0; 2.3]	2.5 [1.8; 2.7]*	2.1 [1.4; 2.2]
SCL-90 Phobic Anxiety	1.6 [1.0; 1.7]*	1.0 [0.6; 1.4]	1.3 [0.7; 1.6]	0.9 [0.6; 1.6]
SCL-90 Paranoid Ideation	2.3 [1.5; 2.5]	2.0 [1.3; 2.5]	2.5 [1.7; 2.7]	2.1 [1.5; 2.7]
SCL-90 Psychoticism	1.8 [1.1; 2.2]*	1.2 [0.5; 1.9]	1.7 [1.3; 2.1]	1.2 [1.0; 1.9]

CSII: Continuous subcutaneous insulin infusion; MDI: Multiple daily injections; HbA1c: Hemoglobin A1c; SCL-90: Symptom Checklist 90; GSI: General Symptomatic Index; PST: Positive Symptom Total; PSDI: Positive Symptom Distress Index. Data are expressed as mean ± SD or median [quartiles]. *P < 0.05 and **P < 0.01 versus CSII.
